# Editorial: Pig translational model in immunological research

**DOI:** 10.3389/fimmu.2024.1456470

**Published:** 2024-07-08

**Authors:** Alla Splichalova, Hauke Smidt, Hirohide Uenishi, Igor Splichal

**Affiliations:** ^1^ Laboratory of Gnotobiology, Institute of Microbiology, Czech Academy of Sciences, Novy Hradek, Czechia; ^2^ Laboratory of Microbiology, Wageningen University & Research, Wageningen, Netherlands; ^3^ Institute of Agrobiological Sciences, National Agriculture and Food Research Organization (NARO), Tsukuba, Japan

**Keywords:** pig, microbiota, swine leukocyte antigen, milk oligosaccharides, toll-like receptor 4, PRRS virus, germ-free

The pig is, due to its similarity to humans in anatomy, physiology, and genetics, a frequently used translational animal model in biomedical research (1). Closed composition of human and pig microbiomes (2, 3) predestine the pig to microbiota-host interference studies. Histological differences in human hemochorial and pig epitheliochorial placentas (4) allow modulation of immunocompetence in newborn piglets reared in microbiologically controlled (gnotobiotic) conditions (5).

Piglets are born without the mother’s protective antibodies that would prevent them from developing sepsis when they are colonized with myriads of microorganisms after birth in conventional conditions. In contrast to prenatal placental transfer of immunoglobulins in infants, newborn piglets obtain protective antibodies after delivery via colostrum uptake (6, 7). Proper colonization of the gastrointestinal tract with microbiota and adequate nutrition are preconditions of thriving piglets in this critical early postnatal period. Choudhury et al. monitored microbiota (16S rRNA gene amplicon sequencing) and transcriptome (RNA sequencing) influenced by customized fibrous feed in piglets from five days after birth until their weaning at 29 days of age. Early feeding accelerated microbiota and transcriptome development to more mature patterns and made piglets more resistant to post-weaning stress, as it was apparent several days after weaning.

The small size of minipigs is convenient regarding housing and manipulation with animals. Goettingen minipigs belong to the most frequently used small pig strains in experimental work (8). Hammer et al. assessed the SLA gene diversity in Goettingen Minipigs in combination with comparative metadata analysis. SLA class I (SLA-1, SLA-2, SLA-3) and class II (DRB1, DQB1, DQA) genes were characterized by PCR-based low-resolution (Lr) haplotyping. Goettingen Minipigs share only six SLA class I and two SLA class II haplotypes with commercial pig lines. Despite the limited number of SLA class I haplotypes, the high genotype diversity being observed necessitates a pre-experimental SLA background assessment of Goettingen Minipigs in regenerative medicine, allotransplantation, and xenograft research. Pernold et al. compared reactivity of peripheral blood mononuclear cells (PMBCs) between humans and Goettingen minipigs, by using a flow cytometry-based *in vitro* proliferation assay, focusing on the T-cell response to various stimuli: concanavalin A (ConA), phytohemagglutinin-L (PHA-L), and staphylococcal Enterotoxin B (SEB). These stimuli were combined with four immunosuppressive drugs: abatacept, belatacept, rapamycin, and tofacitinib. CD4+T cells were more activated in humans, whereas CD8+T cells were generally more abundant in swine. The effectiveness of the used humanized antibodies is most likely related to the conserved structure of CTLA-4, as abatacept induced a much more substantial reduction in swine than belatacept. Treatment with all four compounds resulted in an apparent reduction of the proliferative response, and the drug’s suppressive effectiveness was highly dependent on the stimuli used, mainly in the cases of rapamycin and tofacitinib.

Colostrum and breast milk contain bioactive compounds with anti-microbial, anti-inflammatory, and immunomodulatory properties that contribute to newborn resistance against allergies, asthma, autoimmune diseases, and inflammatory bowel disease (9). Monaco et al. divided 48 h old piglets into four groups and fed them with: (i) sow milk replacer formula (CON), (ii) CON + 6.5 g/L sialylated bovine milk oligosaccharides (BMOS), (iii) human milk oligosaccharides with 1 g/L fucosylated + 0.5 g/L lacto-N-neotetraose neutral milk oligosaccharides (HMO), or (iv) CON+BMOS+HMO. On the 33^rd^ day of postnatal life, serum IgG was significantly lower in the HMO group than BMOS+HMO but comparable with CON or BMOS. The percentage of PBMC T-helper cells was lower in BMOS+HMO than in the other groups. Splenocytes from the BMOS group secreted more IL-1β when stimulated *ex vivo* with LPS than CON or HMO groups. The mixed fucosylated and sialylated oligosaccharides provide specific activities in the immune system that differ from formulations supplemented with one type of oligosaccharides.

The toll-like receptor (TLR) 4 pathway is the primary signaling pathway for the Gram-negative cell wall component lipopolysaccharide (LPS) (10). Li et al. exposed porcine PBMCs to LPS. An Ala610Val variant of glucocorticoid receptor (GR) upregulated a panel of TLR4, its related genes, other pattern recognition receptor genes, cell death, and lymphocyte signaling, ultimately amplifying the inflammatory response. In contrast, dexamethasone pretreatment alleviated the variant orchestrated several genes involved in anti-inflammatory responses. Thus, GR modulation can alleviate inflammatory response in endotoxemia.

Surgically derived colostrum-deprived piglets reared in germ-free (GF) conditions (5) allow the evaluation of the impact of specific microbes on the host without a background of non-identified microbiota. Stepanova et al. found that the production of pro-inflammatory cytokine IL-17A in PMA and ionomycin-stimulated blood, spleen, and mesenteric lymph nodes (MLN)-derived cells from conventional piglets were significantly higher in blood, spleen, and MLN for CD3+CD4+ cells and in spleen and MLN for CD3+TCRγδ+ cells than in their GF counterparts. IL-17A production in GF piglets was negligible and without any age-dependent progress.

Porcine reproductive and respiratory syndrome virus (PRRSV) emerged more than 30 years ago in the US and almost simultaneously but independently in Europe (11). Stepanova et al. tested three modified live vaccine (MLV) PRRSV strains and compared their effect to the wild virus type 1 or 2. MLV strains cause depletion of T-cell precursors, alteration of the TCR repertoire, necrobiosis at corticomedullary junctions, decreased thymic cellularity, lack of virus-neutralizing antibodies, production of non-neutralizing anti-PRRSV antibodies of different isotypes, and low body weight gain comparable to wild-type PRRSV. Thus, their use can be dangerous for young piglets.

Progress in xenotransplantation of pig organs to humans underlines the importance of the pig in biomedical research (12). Therefore, further development and characterization of pig models are required, and new knowledge in this area is welcomed.

## Author contributions

AS: Writing – original draft, Writing – review & editing. HS: Writing – review & editing. HU: Writing – review & editing. IS: Writing – original draft, Writing – review & editing.
